# TaF_4_: A Novel Two-Dimensional Antiferromagnetic Material with a High Néel Temperature Investigated Using First-Principles Calculations

**DOI:** 10.3390/ma17112780

**Published:** 2024-06-06

**Authors:** Jia Luo, Qingkai Zhang, Jindong Lin, Yuxiang Ni, Hongyan Wang, Yongliang Tang, Mu Lan

**Affiliations:** 1 Key Laboratory of Advanced Technologies of Materials, Ministry of Education of China, School of Physical Science and Technology, Southwest Jiaotong University, Chengdu 610032, China; 2 College of Optoelectronic Engineering, Chengdu University of Information Technology, Chengdu 610103, China

**Keywords:** two-dimensional material, TaF_4_, first-principles calculations, high Néel temperature

## Abstract

The structural, electronic, and magnetic properties of a novel two-dimensional monolayer material, TaF_4_, are investigated using first-principles calculations. The dynamical and thermal stabilities of two-dimensional monolayer TaF_4_ were confirmed using its phonon dispersion spectrum and molecular dynamics calculations. The band structure obtained via the high-accuracy HSE06 (Heyd–Scuseria–Ernzerhof 2006) functional theory revealed that monolayer two-dimensional TaF_4_ is an indirect bandgap semiconductor with a bandgap width of 2.58 eV. By extracting the exchange interaction intensities and magnetocrystalline anisotropy energy in a *J*_1_-*J*_2_-*J*_3_-*K* Heisenberg model, it was found that two-dimensional monolayer TaF_4_ possesses a Néel-type antiferromagnetic ground state and has a relatively high Néel temperature (208 K) and strong magnetocrystalline anisotropy energy (2.06 meV). These results are verified via the magnon spectrum.

## 1. Introduction

Two-dimensional (2D) antiferromagnetic (AFM) materials, which have traditionally been emphasized less in spintronics compared to two-dimensional ferromagnetic materials, have gained significant attention in recent years due to their atomic arrangement, structural characteristics, and exceptional physical properties [1,2,3,4]. Their magnetic moments are arranged anti-parallel in a two-dimensional plane, forming an ordered magnetic structure [5]. Characterized by an anti-parallel alignment of adjacent valence electron spins, these materials exhibit no net macroscopic magnetization, avoiding stray magnetic fields [1]. This feature facilitates high-frequency spin dynamics, positioning antiferromagnets as promising materials for next-generation high-speed and high-density spintronics [1,2,6,7,8,9,10,11,12,13].

In recent years, significant progress has been made in the study of two-dimensional antiferromagnetic materials. For example, the anomalous Hall effect and the antiferromagnetic-spin Hall effect in collinear antiferromagnetism were confirmed [14,15]. Researchers rely on magnetic fields, electric fields, light fields, external pressure, and other methods to control the antiferromagnetic sequence [16,17,18]. Efforts have also been made to manipulate spin and magnetism in two-dimensional limits for efficient applications of two-dimensional materials [19,20,21,22,23,24]. However, although some important results have been achieved, many problems remain to be solved, such as determining how to precisely control growth conditions and parameters to prepare high-quality, uniform two-dimensional antiferromagnetic materials with large areas, how to further improve the stability of two-dimensional antiferromagnetic materials, how to precisely regulate their magnetic properties, and how to use them effectively.

Two-dimensional tetrafluorides have become a research hotspot due to the low level of lattice symmetry and band splitting in their antiferromagnetic ordering. Novel 2D tetrafluoride structures such as VF_4_, MnF_4_, and RuF_4_ have been successively reported [25,26,27]. In this study, we utilized first-principles calculations based on the density functional theory (DFT) to predict a novel 2D monolayer antiferromagnetic material—TaF_4_. It exhibits both dynamical and thermodynamical stability. Through an investigation of its structural, electrical, and magnetic properties, we found it possesses a Néel-type antiferromagnetic ground state and has a relatively high Néel temperature. These results suggest its potential application as a novel 2D antiferromagnetic material in spintronics.

## 2. Methods

Spin-polarized first-principles calculations were performed within the density functional theory framework, which we implemented in the Vienna Ab-initio Simulation Package (VASP 5.4.1) [28,29]. The electron configurations selected in the calculation were Ta(5*p*^6^ 5*d*^3^ 6*s*^2^) and F(2*s*^2^ 2*p*^5^), the core electrons were treated within projector-augmented wave (PAW) pseudopotentials [30], the exchange and correlation interactions between electrons were described through the generalized gradient approximation (GGA) of the revised Perdew, Burke, and Ernzerhof functional for solids (PBEsol) [31,32]. The kinetic energy cutoff was set to 520 eV, electronic energy minimization was performed with a tolerance of 1.0 × 10^−7^ eV, and the convergence criterion for the force on each atom was set to 0.001 eV/Å. Brillouin zone (BZ) sampling was performed using a 7 × 7 × 1 grid for unit cell relaxation calculations and a 9 × 9 × 1 grid for static calculations. A vacuum larger than 22 Å was applied to avoid an interaction between the monolayers caused by the periodic boundary condition. In particular, we used the HSE06 (Heyd–Scuseria–Ernzerhof 2006) hybrid functional including a 25% non-local Hartree–Fock exchange to correct the underestimated bandgaps and achieve a more accurate prediction of the electronic structure and the energies of the magnetic states [33]. Phonon dispersion was calculated using the density functional perturbation theory, which was implemented in the PHONOPY (2.22.1) package [34,35], to check the dynamical stability of the TaF_4_. The thermal stability of the structure was tested by Ab-initio molecular dynamics simulations at 500 K for 1000 steps with a time step of 2 fs. Monte Carlo (MC) simulations were performed in 80 × 80 × 1 TaF_4_ supercell structures using mcsolver (21.03.08) [36].

## 3. Results and Discussion

**Figure 1:**
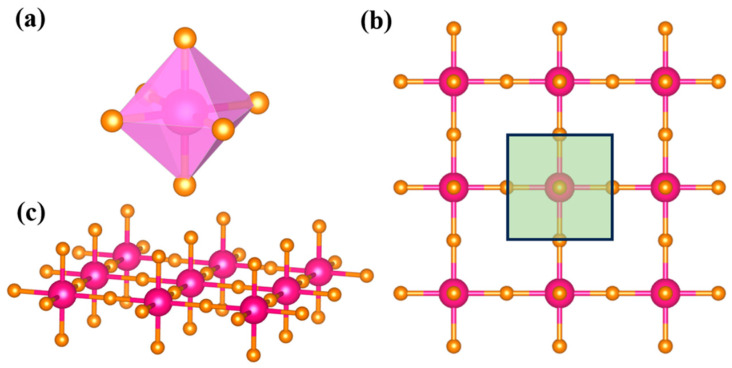
Unit cell (**a**), top (**b**), and side (**c**) views of atomic structure of 2D TaF4. Magenta and orange balls represent Ta and F atoms, respectively.

With a sufficiently large vacuum layer (22 Å), by relaxing the lattice structure to achieve sufficiently low system energy and meet the force convergence criteria, the lattice constants after structural optimization can be obtained. The optimized geometric structure of the 2D TaF_4_ under consideration is presented in Figure 1. The geometry of the 2D TaF_4_ is composed of three atomic planes: the two layers of F atoms are symmetrically distributed on both sides of the middle layer composed of Ta and F atoms, which is different to the well-known transition-metal dichalcogenides (TMDCs). Obviously, each Ta atom is surrounded by six neighboring F atoms, forming six Ta–F bonds and an octahedron. We determined the stable and minimum energy structures by optimizing all atomic positions and lattice constants. The optimized bond lengths of the 2D TaF_4_ are 2.05 Å (parallel to the plane) and 1.87 Å (perpendicular to the plane).

**Figure 2:**
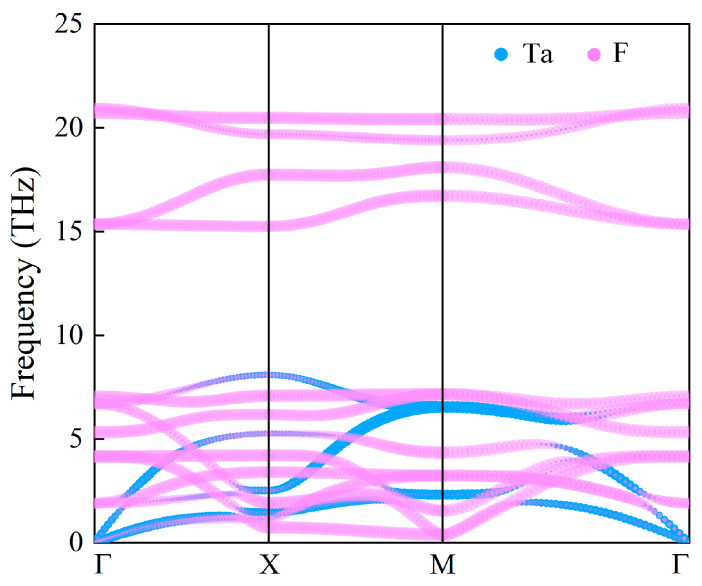
The phonon dispersion of the 2D TaF_4_. Blue (pink) lines represent the contribution of the Ta (F) atoms’ vibration.

Before studying the electric and magnetic properties of the 2D TaF_4_, its dynamical stability and thermodynamical stability should be verified first. Figure 2 illustrates the phonon spectrum of the 2D TaF_4_. No appreciable imaginary frequencies are found in the whole Brillouin zone, which confirms that the TaF_4_ monolayers are dynamically stable. The phonon dispersion of the TaF_4_ contains 3 acoustic branches and 12 optical branches as one unit cell contains one Ta atom and four F atoms. It can be observed that the contribution of the vibration of the Ta atoms is mainly concentrated in low-energy phonon bands, while the high-energy phonon bands are primarily formed by the vibration of F atoms. As the lattice structure belongs to the space group P4/mmm, the 2D TaF_4_ exhibits higher symmetry than other 2D tetrafluoride compounds, such as VF_4_, MnF_4_, and RuF_4_ [25,26,27]. As shown in Table 1, the vibration modes of the optical branches in the long-wave limit could manifest as Raman activity, which is detectable through Raman spectroscopy, infrared (IR) activity, observable via IR spectroscopy, or they could be silent. The vibration modes of the 2D TaF_4_ monolayer can be visualized in Supplementary Materials S1. One can also notice the existence of a considerable phonon band gap, spanning approximately from 8 GHz to 15 GHz, within the optical branches. This indicates that 2D TaF_4_ could be used as a low-dimensional thermal resistance material because the introduction of a phonon gap can significantly reduce thermal conductivity.

Ab initio molecular dynamics simulations were performed to investigate thermodynamical stability; they were performed at an ambient temperature for 1000 steps with a time step of 2 fs. After the molecular dynamics simulations, the structure was slightly rippled but remained intact and did not collapse; this confirmed the thermodynamical stability of the 2D TaF_4_ monolayer (Supplementary Material S2).

**Figure 3:**
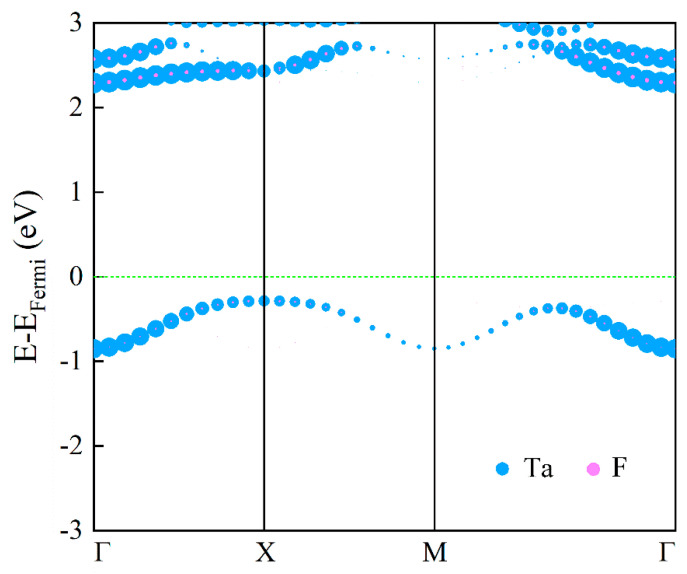
The spin-polarized band structure of the 2D TaF_4_ monolayer calculated with the high-accuracy HSE06 functional. The horizontal dotted line indicates the Fermi energy level.

In Figure 3, we depict the electronic band structure of the antiferromagnetic state of 2D TaF_4_ in the first Brillouin zone, which was unfolded from the band of the AFM state in a $\sqrt{2}\times \sqrt{2}$ cell. Due to the degeneration of two spin channels in the AFM state, we do not display the spin-up and spin-down bands separately while including spin–orbit coupling. The blue and pink lines represent the contributions of the Ta and F atoms, respectively. With the HSE06 hybrid functional theory, one can obviously note that no energy band passes through the Fermi level, and the valence band maximum (VBM) is located at the X point, while the conduction band minimum (CBM) is located at the Γ point. Thus, the 2D TaF_4_ is an indirect bandgap semiconductor with a bandgap of about 2.58 eV.

**Figure 4:**
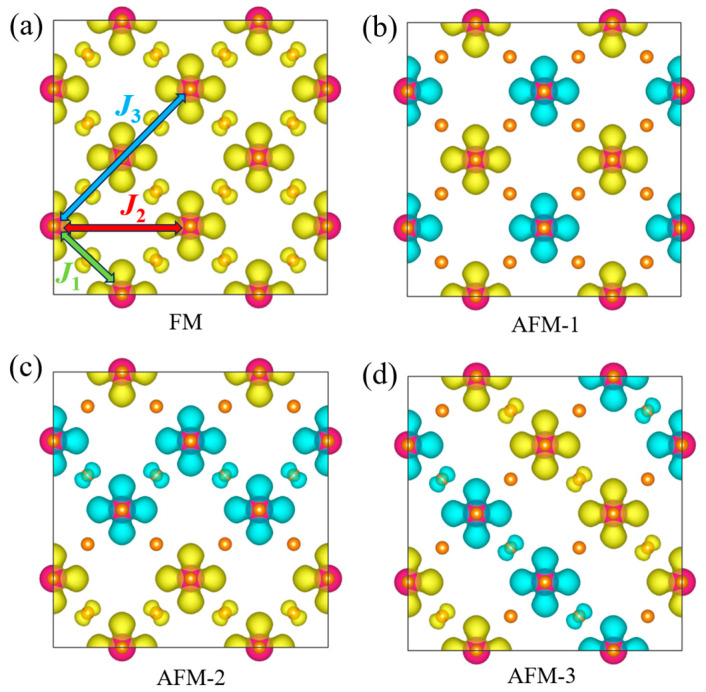
Spin density isosurfaces of four different-magnetic-order structures: (**a**) FM, (**b**) AFM-1, (**c**) AFM-2, and (**d**) AFM-3. Yellow and blue isosurfaces indicate spin-up and spin-down densities, respectively. Exchange interactions of nearest-neighbor *J*_1_, next nearest-neighbor *J*_2_, and third nearest-neighbor *J*_3_ are pointed out in figure (**a**) with arrows.

In order to discuss the magnetic properties of the 2D TaF_4_, we compared the energies of a ferromagnetic (FM) order (shown in Figure 4a) and three different AFM orders (shown in Figure 4b–d) to confirm the ground state of the monolayer 2D TaF_4_. As a result, the 2D TaF_4_ prefers to adopt a Néel-type AFM order, labeled AFM-1 in Figure 4b. To discuss the magnetic stability of the 2D TaF_4_, we need to determine the exchange interaction intensities between the atoms in the monolayer. From Figure 4, we can see that little spin charge pervades near the F atoms, while the spin density is mainly concentrated on the Ta atoms. Thus, we could omit the existence of the F atoms, and simplify the magnetic lattice of the 2D TaF_4_ into a *J*_1_-*J*_2_-*J*_3_-*K* Heisenberg model in a square lattice where the *J*_1_, *J*_2_, and *J*_3_ represent the nearest, next-nearest, and third-nearest exchange interactions, respectively. Comparing the energy difference in the FM and three different AFM orders of this system, we can extract the exchange interactions between Ta atoms. For each type of magnetic order, we have the following relationships:

*E*_FM_ = *E*_0_ − 16*J*_1_*S*^2^ − 16*J*_2_*S*^2^ − 16*J*_3_*S*^2^ (1)

*E*_AFM-1_ = *E*_0_ + 16*J*_1_*S*^2^ − 16*J*_2_*S*^2^ − 16*J*_3_*S*^2^ (2)

*E*_AFM-2_ = *E*_0_ + 16*J*_3_*S*^2^ (3)

*E*_AFM-3_ = *E*_0_ + 16*J*_2_*S*^2^ − 16*J*_3_*S*^2^ (4)

where *E*_0_ represents energy without considering spin, and *S* represents the spin on Ta atoms. After bonding with six F atoms, the tetravalent Ta has one 5*d* electron left unbonded. Thus, the Ta atom shows spin with *S* = 1/2. The calculated exchange parameters *J*_1_, *J*_2_, and *J*_3_ are −92.00 meV, −1.48 meV, and −3.28 meV, respectively. The negative values of the exchange interactions prefer to spin antiparallel to two Ta atoms. As *J*_1_ is apparently stronger than *J*_2_ and *J*_3_, the nearest exchange dominates the spin arrangement. Thus, we can deduce that without thermal fluctuation, the spin of a Ta atom should align oppositely with its four nearest Ta atoms, which have the same spin orientation. As a result, the ground state of the Néel-type AFM order of the 2D TaF_4_ should be stable at low temperatures.

**Figure 5:**
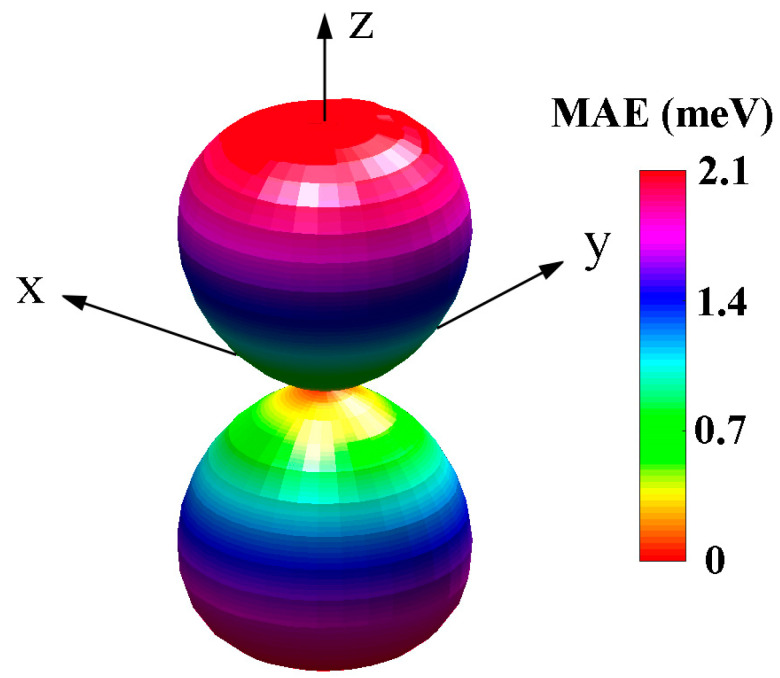
Magnetocrystalline anisotropy energy of monolayer 2D TaF_4_.

As the Ta atom is relatively heavy, its existence could lead to significant spin–orbit coupling interactions, which might lead to a large magnetocrystalline anisotropy energy (MAE). Thus, we can evaluate the MAE as described in the formula below:

(5) $$K=\frac{E_{in-plane}-E_{tilt}}{S^{2}\cos^{2}\left(\theta \right)}$$

where $E_{in-plane}$ and $E_{tilt}$ represent energy with a magnetic moment parallel to the plane and inclined to the plane with θ degrees. As shown in Figure 5, it can be seen that the anisotropic energy results in the 2D TaF_4_ having an out-of-plane easy axis, which indicates that the magnetic moments tend to be perpendicular to the plane of the monolayer. In the z direction perpendicular to the plane is the maximum magnetocrystalline anisotropy energy of about 2.06 meV, this value is much larger than those in MnX_2_ and GdX_2_ [37,38].

**Figure 6:**
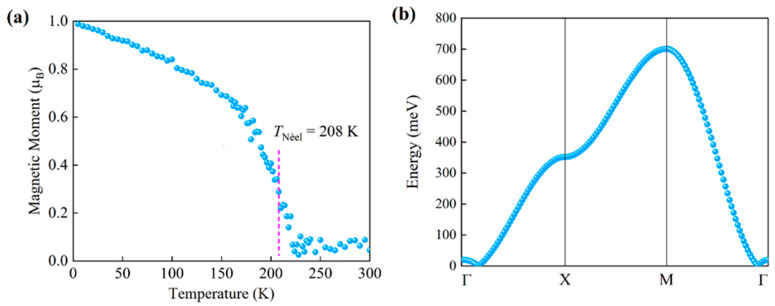
Temperature dependence of magnetic moment (**a**) and Magnon spectra (**b**) of 2D TaF_4_.

With the strong AFM nearest the exchange interactions and MAE, we can imagine that the 2D TaF_4_ could possess a stable Néel-type AFM order. Based on the *J*_1_-*J*_2_-*J*_3_-*K* Heisenberg model, we evaluated the antiferromagnetic-to-paramagnetic phase transition temperature, namely the Néel temperature (T_N_), using Monte Carlo simulations. Based on the calculated exchange interactions and magnetocrystalline anisotropy intensities, Monte Carlo simulations were carried out, conducting 200,000 steps at different temperatures on an 80 × 80 supercell of monolayer TaF_4_. The magnetic moments of the Ta atoms in the 2D TaF_4_ can be variously influenced by thermal fluctuations depending on the varying temperatures. The temperature dependence of the average magnetic moment on one sublattice of Ta atoms in the 2D TaF_4_ AFM order is shown in Figure 6a. We can notice that the magnetic moment on the Ta atom in the TaF_4_ monolayer gradually drops as the temperature rises. The point with the highest slope during the moment decrease corresponds to the T_N_ of the structure. At this temperature, the antiferromagnetic order of the structure disappears, and the structure undergoes a transition from an antiferromagnetic state to a paramagnetic state. It can be seen that the calculated T_N_ of the TaF_4_ monolayer is about 208 K. This indicates that the monolayer TaF_4_ might be a good candidate as a 2D material for spintronic and electronic applications.

Using the exchange interactions we obtained and based on the Landau–Lifshitz–Gilbert equations, the magnon dispersion of the monolayer TaF_4_ was studied with the atomistic spin dynamics approach. As shown in Figure 6b, it can be seen that one acoustic branch of magnon in the dispersion is found while no optical branch is observed because only one TaF_4_ layer is involved. The magnon curve of the monolayer TaF_4_ satisfies a nearly linear relationship near the Γ point, indicating that the monolayer TaF_4_ possesses an antiferromagnetic state. The nonzero energy at the Γ point corresponds to the strong MAE. The magnon energy at the Brillouin zone boundary is relatively large compared with other two-dimensional materials and the local maximum magnon energy of the monolayer TaF_4_ is about 700 meV at M point, which implies the strong stability of its Néel-type AFM order.

**Figure 7:**
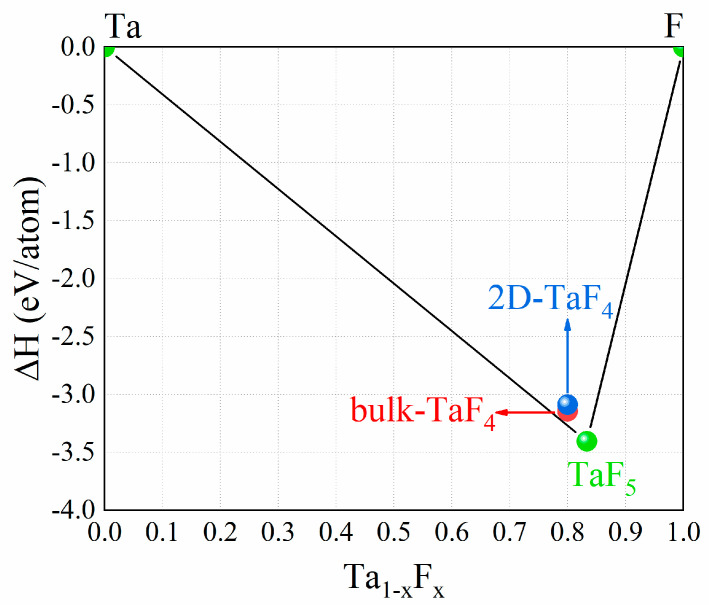
Formation energy of 2D TaF_4_ in convex hull of Ta-F compounds.

Lastly, to consider the experimental feasibility of the 2D TaF_4_, we calculated its formation energy and interlayer binding energy. Figure 7 shows that the formation energy of the 2D TaF_4_ is 0.056 eV/atom higher than its bulk counterpart and 0.180 eV/atom above the convex hull. These minimal energy differences suggest that the 2D TaF_4_ is very likely to exist in a metastable form [39]. Additionally, we calculated its interlayer binding energy. The calculated interlayer binding energy of the 2D TaF_4_ is about 17.68 meV/Å^2^, which is not much higher than that of graphene (11.83 meV/Å^2^) [40], indicating that 2D TaF_4_ could potentially be obtained through mechanical exfoliation.

## 4. Conclusions

In summary, a novel monolayer 2D material, TaF_4_, was investigated using first-principles methods. Its dynamical stability and thermodynamic stability were confirmed via phonon spectrum and ab initio molecular dynamics simulations. Employing the high-accuracy HSE06 functional theory, we determined that 2D TaF_4_ is an indirect bandgap semiconductor with a bandgap of 2.58 eV. Based on the Heisenberg model, by comparing the energy differences between different magnetic orders, we obtained the exchange interaction intensities between Ta atoms and the magnetic anisotropy of the system. The strong AFM nearest-neighbor exchange interaction and MAE illustrate that the 2D TaF_4_ could possess a stable Néel-type AFM order at low temperatures, and a relatively high Néel temperature (208 K) was predicted. The antiferromagnetic ground state and strong magnetic crystalline anisotropy of the 2D TaF_4_ were verified by its magnon spectrum. Through calculations of its formation energy and interlayer binding energy, we speculate that the 2D TaF_4_ has the possibility to be obtained through mechanical exfoliation. This work suggests that TaF_4_ could serve as a low-dimensional thermal resistance material and might be a promising candidate for spintronic and electronic applications.

## Figures and Tables

**Table 1:** Energy (unit: THz), irreducible representations (Irreps.), and activity (R for Raman and IR for infrared) of long-wave-limit phonons.

E (THz)	Irreps.	Activity
0	E_u_	-
0	A_2u_	-
1.904	E_u_	IR
4.159	E_g_	R
5.327	B_2u_	-
6.706	E_u_	IR
7.081	A_2u_	IR
15.351	E_u_	IR
20.727	A_1g_	R
20.967	A_2u_	IR

## Data Availability

The original contributions presented in the study are included in the article/Supplementary Material, further inquiries can be directed to the corresponding authors.

## Supplementary Materials

The following supporting information can be downloaded at https://www.mdpi.com/article/10.3390/ma17112780/s1. See the Supplementary Materials for the vibration modes and molecular dynamics simulations of the 2D TaF_4_ monolayer. Supplementary Material S1—Vibration modes of the 2D TaF4. Supplementary Material S2—Ab initio molecular dynamics simulations.

## References

[1] Jungwirth T., Marti X., Wadley P., Wunderlich J. (2016). Antiferromagnetic spintronics. Nat. Nanotechnol..

[2] Železný J., Wadley P., Olejník K., Hoffmann A., Ohno H. (2018). Spin transport and spin torque in antiferromagnetic devices. Nat. Phys..

[3] Chen H., Liu L., Zhou X., Meng Z., Wang X., Duan Z., Zhao G., Yan H., Qin P., Liu Z. (2024). Emerging Antiferromagnets for Spintronics. Adv. Mater..

[4] Araujo R.d.M.T., Zarpellon J., Mosca D.H. (2022). Unveiling ferromagnetism and antiferromagnetism in two dimensions at room temperature. J. Phys. D Appl. Phys..

[5] Hao L., Meyers D., Suwa H., Yang J., Frederick C., Dasa T.R., Fabbris G., Horak L., Kriegner D., Choi Y. (2018). Giant magnetic response of a two-dimensional antiferromagnet. Nat. Phys..

[6] Ke J., Yang M., Xia W., Zhu H., Liu C., Chen R., Dong C., Liu W., Shi M., Guo Y. (2020). Magnetic and magneto-transport studies of two-dimensional ferromagnetic compound Fe_3_GeTe_2_. J. Phys. Condens. Matter.

[7] Meier F., Levy J., Loss D. (2003). Quantum computing with antiferromagnetic spin clusters. Phys. Rev. B.

[8] Roscilde T., Verrucchi P., Fubini A., Haas S., Tognetti V. (2005). Entanglement and factorized ground states in two-dimensional quantum antiferromagnets. Phys. Rev. Lett..

[9] Wadley P., Howells B., Železný J., Andrews C., Hills V., Campion R.P., Novák V., Olejník K., Maccherozzi F., Dhesi S. (2016). Electrical switching of an antiferromagnet. Science.

[10] Li X., Yang J. (2016). First-principles design of spintronics materials. Natl. Sci. Rev..

[11] Baltz V., Manchon A., Tsoi M., Moriyama T., Ono T., Tserkovnyak Y. (2018). Antiferromagnetic spintronics. Rev. Mod. Phys..

[12] Lavrijsen R. (2019). A new twist for spin torques in antiferromagnets. Nat. Electron..

[13] Gu P., Wang C., Su D., Dong Z., Wang Q., Han Z., Watanabe K., Taniguchi T., Ji W., Sun Y. (2023). Multi-state data storage in a two-dimensional stripy antiferromagnet implemented by magnetoelectric effect. Nat. Commun..

[14] Feng Z., Zhou X., Šmejkal L., Wu L., Zhu Z., Guo H., González-Hernández R., Wang X., Yan H., Qin P. (2022). An anomalous Hall effect in altermagnetic ruthenium dioxide. Nat. Electron..

[15] Šmejkal L., González-Hernández R., Jungwirth T., Sinova J. (2020). Crystal time-reversal symmetry breaking and spontaneous Hall effect in collinear antiferromagnets. Sci. Adv..

[16] Gao Y., Jiang X., Qiu Z., Zhao J. (2023). Photoexcitation induced magnetic phase transition and spin dynamics in antiferromagnetic MnPS3 monolayer. NPJ Comput. Mater..

[17] He J., Li S., Frauenheim T., Zhou Z. (2023). Ultrafast Laser Pulse Induced Transient Ferrimagnetic State and Spin Relaxation Dynamics in Two-Dimensional Antiferromagnets. Nano Lett..

[18] Li S., Zhou L., Frauenheim T., He J. (2022). Light-Controlled Ultrafast Magnetic State Transition in Antiferromagnetic–Ferromagnetic van der Waals Heterostructures. J. Phys. Chem. Lett..

[19] Lee J.-U., Lee S., Ryoo J.H., Kang S., Kim T.Y., Kim P., Park C.-H., Park J.-G., Cheong H. (2016). Ising-type magnetic ordering in atomically thin FePS3. Nano Lett..

[20] Kim K., Lim S.Y., Lee J.-U., Lee S., Kim T.Y., Park K., Jeon G.S., Park C.-H., Park J.-G., Cheong H. (2019). Suppression of magnetic ordering in XXZ-type antiferromagnetic monolayer NiPS3. Nat. Commun..

[21] Long G., Zhang T., Cai X., Hu J., Cho C.-W., Xu S., Shen J., Wu Z., Han T., Lin J. (2017). Isolation and characterization of few-layer manganese thiophosphite. ACS Nano.

[22] Deng Y., Yu Y., Song Y., Zhang J., Wang N.Z., Sun Z., Yi Y., Wu Y.Z., Wu S., Zhu J. (2018). Gate-tunable room-temperature ferromagnetism in two-dimensional Fe_3_GeTe_2_. Nature.

[23] Huang B., Clark G., Navarro-Moratalla E., Klein D.R., Cheng R., Seyler K.L., Zhong D., Schmidgall E., McGuire M.A., Cobden D.H. (2017). Layer-dependent ferromagnetism in a van der Waals crystal down to the monolayer limit. Nature.

[24] Zhou Y., He K., Hu H., Ouyang G., Zhu C., Wang W., Qin S., Tao Y., Chen R., Zhang L. (2022). Strong Neel Ordering and Luminescence Correlation in a Two-Dimensional Antiferromagnet. Laser Photonics Rev..

[25] Zhang L., Tang C., Du A. (2021). Two-dimensional vanadium tetrafluoride with antiferromagnetic ferroelasticity and bidirectional negative Poisson’s ratio. J. Mater. Chem. C.

[26] Xu S., Jia F., Cheng X., Ren W. (2021). Predicting intrinsic antiferromagnetic and ferroelastic MnF 4 monolayer with controllable magnetization. J. Mater. Chem. C.

[27] Wang N., Chen J., Ding N., Zhang H., Dong S., Wang S.-S. (2022). Magneto-optical Kerr effect and magnetoelasticity in a weakly ferromagnetic RuF 4 monolayer. Phys. Rev. B.

[28] Kresse G., Furthmüller J. (1996). Efficient iterative schemes for ab initio total-energy calculations using a plane-wave basis set. Phys. Rev. B.

[29] Kresse G., Furthmüller J. (1996). Efficiency of ab-initio total energy calculations for metals and semiconductors using a plane-wave basis set. Comput. Mater. Sci..

[30] Blöchl P.E. (1994). Projector augmented-wave method. Phys. Rev. B.

[31] Perdew J.P., Burke K., Ernzerhof M. (1996). Generalized gradient approximation made simple. Phys. Rev. Lett..

[32] Söderlind P., Eriksson O., Johansson B., Wills J. (1994). Electronic properties of f-electron metals using the generalized gradient approximation. Phys. Rev. B.

[33] Heyd J., Scuseria G.E., Ernzerhof M. (2003). Hybrid functionals based on a screened Coulomb potential. J. Chem. Phys..

[34] Togo A., Tanaka I. (2015). First principles phonon calculations in materials science. Scr. Mater..

[35] Baroni S., De Gironcoli S., Dal Corso A., Giannozzi P. (2001). Phonons and related crystal properties from density-functional perturbation theory. Rev. Mod. Phys..

[36] Liu L., Ren X., Xie J., Cheng B., Liu W., An T., Qin H., Hu J. (2019). Magnetic switches via electric field in BN nanoribbons. Appl. Surf. Sci..

[37] Luo J., Xiang G., Tang Y., Ou K., Chen X. (2020). The electric and magnetic properties of novel two-dimensional MnBr2 and MnI2 from first-principles calculations. J. Appl. Phys..

[38] Luo J., Ou K., Tang Y., Zhang W., Ni Y., Wang H., Lan M. (2023). The electric and magnetic properties of novel two-dimensional H and T’Phase GdX2 (X = F, Cl, Br, I) from first-principles calculations. Eur. Phys. J. Plus.

[39] Zurek E. (2016). Discovering new materials via a priori crystal structure prediction. Rev. Comput. Chem..

[40] Liu Z., Liu J.Z., Cheng Y., Li Z., Wang L., Zheng Q. (2012). Interlayer binding energy of graphite: A mesoscopic determination from deformation. Phys. Rev. B.

