# Supplementary figures and images for: TaF4: A Novel Two-Dimensional Antiferromagnetic Material with a High Néel Temperature Investigated Using First-Principles Calculations
Source: Materials (Basel). 2024 Jun 6;17(11):2780. doi: 10.3390/ma17112780 (PMC11173987; doi:10.3390/ma17112780)

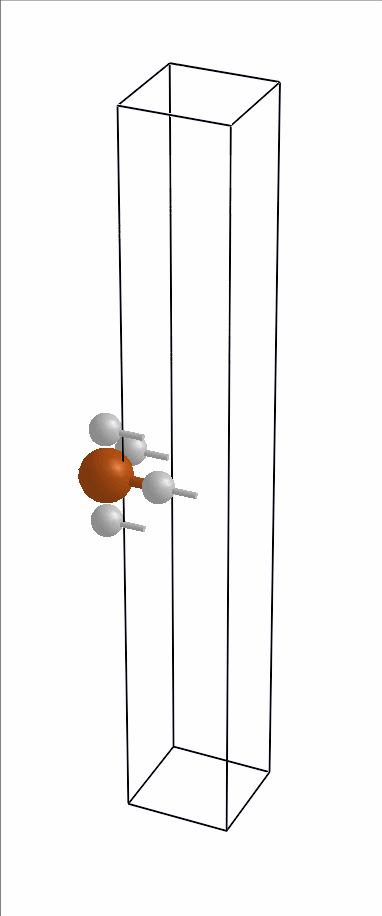

Supplement: Supplementary file 1 [file materials-17-02780-s001.zip › materials-3012524-supplementary/Supplementary Materials/Supplementary material S1 - Vibration modes of 2D TaF4/01.gif]

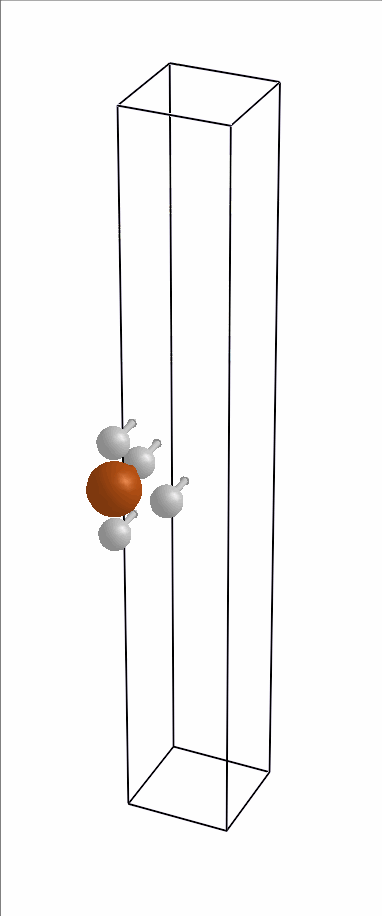

Supplement: Supplementary file 1 [file materials-17-02780-s001.zip › materials-3012524-supplementary/Supplementary Materials/Supplementary material S1 - Vibration modes of 2D TaF4/02.gif]

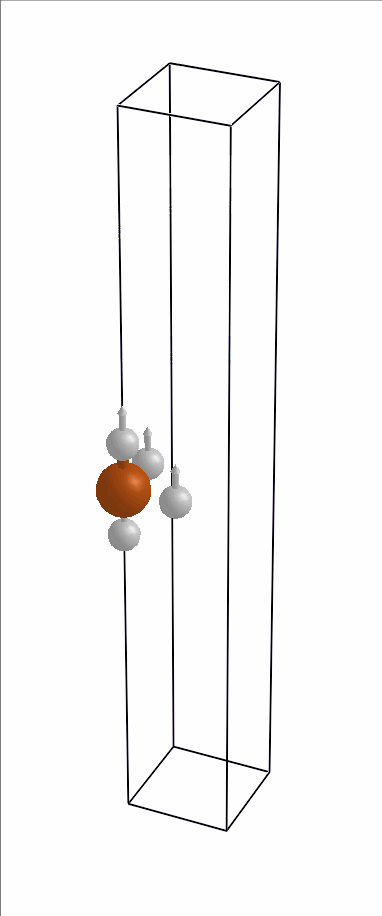

Supplement: Supplementary file 1 [file materials-17-02780-s001.zip › materials-3012524-supplementary/Supplementary Materials/Supplementary material S1 - Vibration modes of 2D TaF4/03.gif]

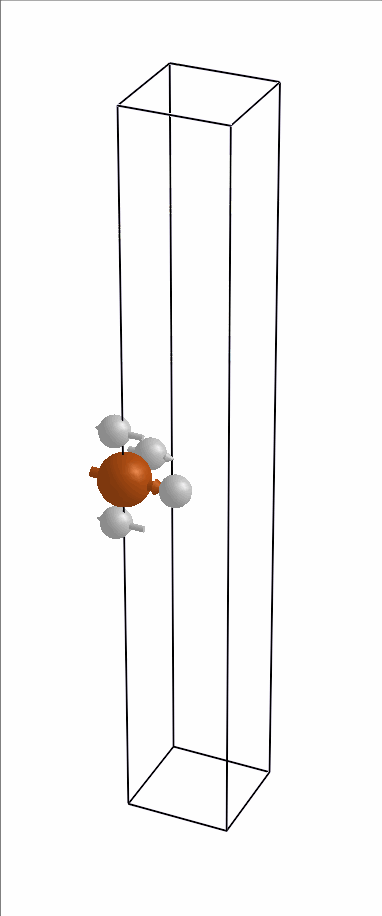

Supplement: Supplementary file 1 [file materials-17-02780-s001.zip › materials-3012524-supplementary/Supplementary Materials/Supplementary material S1 - Vibration modes of 2D TaF4/04.gif]

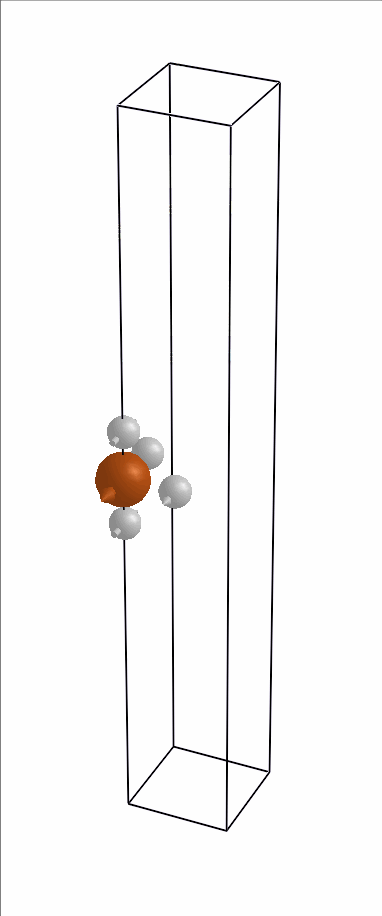

Supplement: Supplementary file 1 [file materials-17-02780-s001.zip › materials-3012524-supplementary/Supplementary Materials/Supplementary material S1 - Vibration modes of 2D TaF4/05.gif]

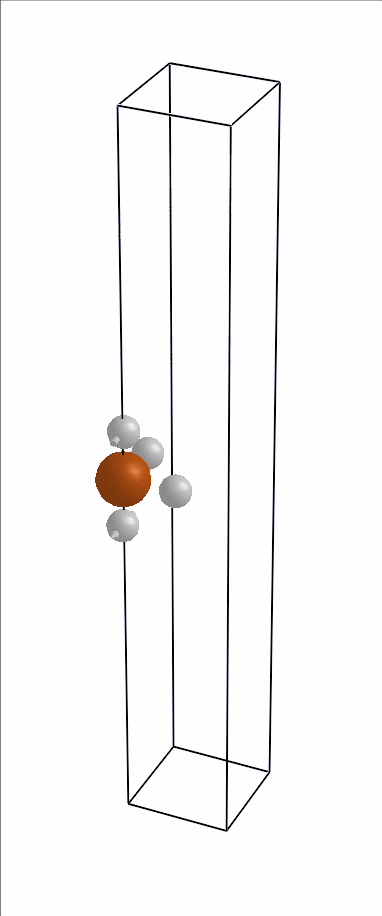

Supplement: Supplementary file 1 [file materials-17-02780-s001.zip › materials-3012524-supplementary/Supplementary Materials/Supplementary material S1 - Vibration modes of 2D TaF4/06.gif]

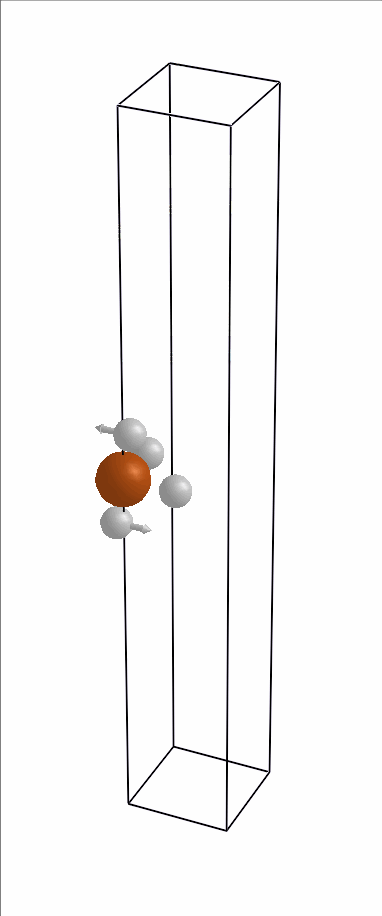

Supplement: Supplementary file 1 [file materials-17-02780-s001.zip › materials-3012524-supplementary/Supplementary Materials/Supplementary material S1 - Vibration modes of 2D TaF4/07.gif]

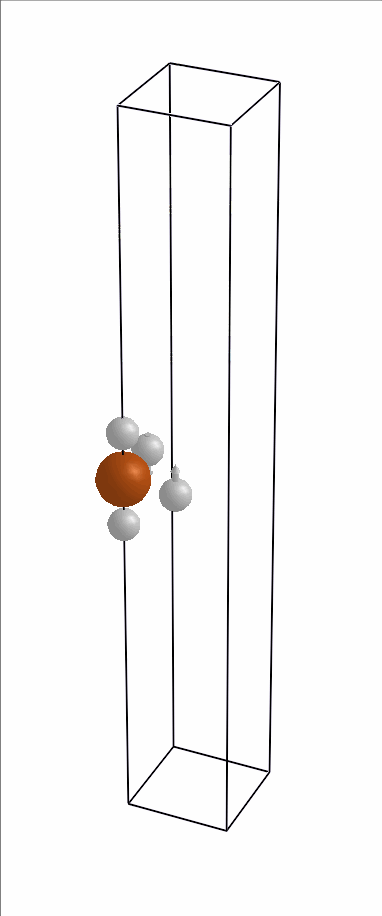

Supplement: Supplementary file 1 [file materials-17-02780-s001.zip › materials-3012524-supplementary/Supplementary Materials/Supplementary material S1 - Vibration modes of 2D TaF4/08.gif]

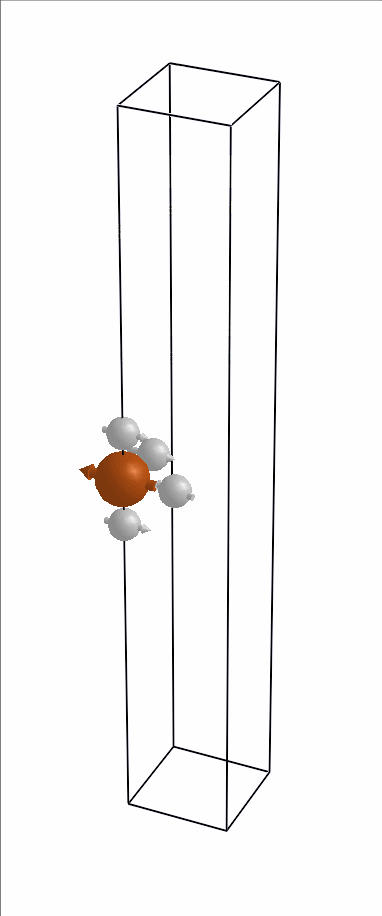

Supplement: Supplementary file 1 [file materials-17-02780-s001.zip › materials-3012524-supplementary/Supplementary Materials/Supplementary material S1 - Vibration modes of 2D TaF4/09.gif]

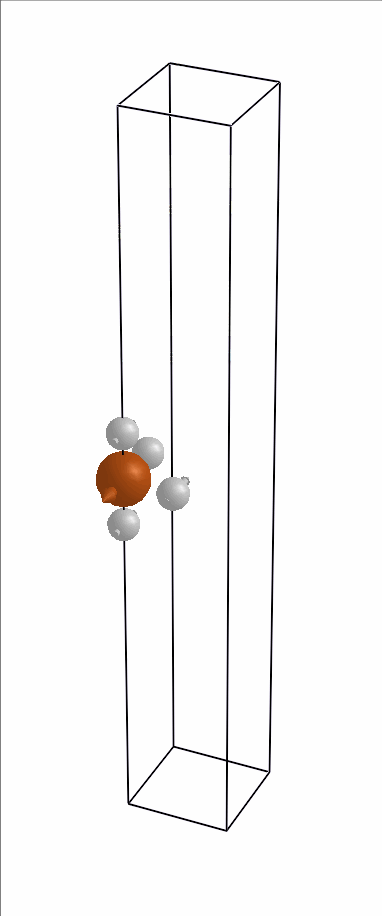

Supplement: Supplementary file 1 [file materials-17-02780-s001.zip › materials-3012524-supplementary/Supplementary Materials/Supplementary material S1 - Vibration modes of 2D TaF4/10.gif]

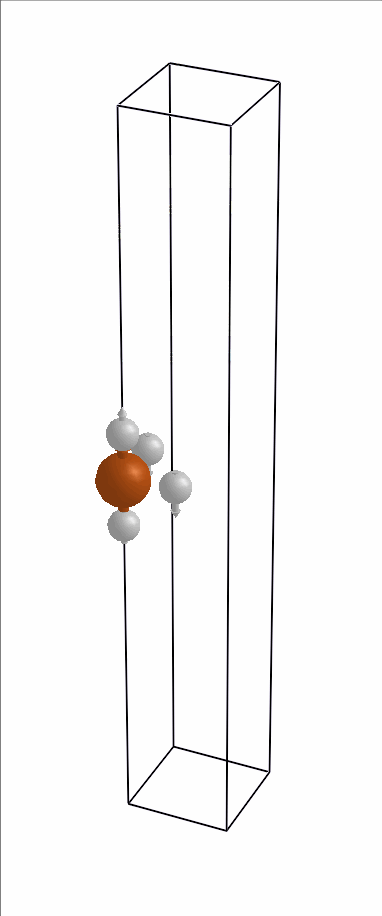

Supplement: Supplementary file 1 [file materials-17-02780-s001.zip › materials-3012524-supplementary/Supplementary Materials/Supplementary material S1 - Vibration modes of 2D TaF4/11.gif]

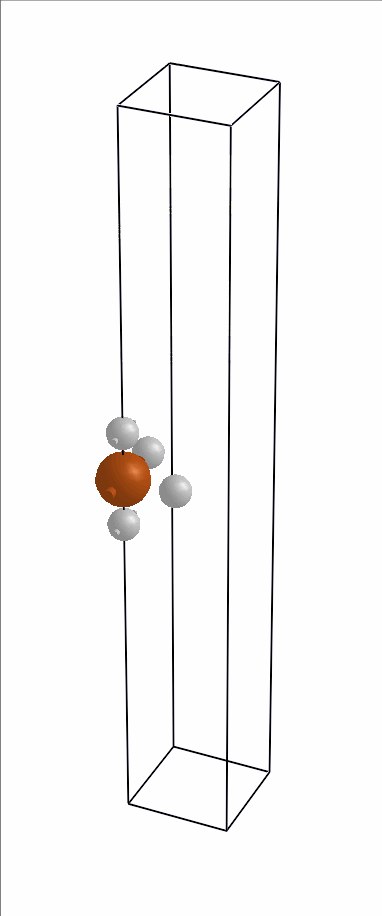

Supplement: Supplementary file 1 [file materials-17-02780-s001.zip › materials-3012524-supplementary/Supplementary Materials/Supplementary material S1 - Vibration modes of 2D TaF4/12.gif]

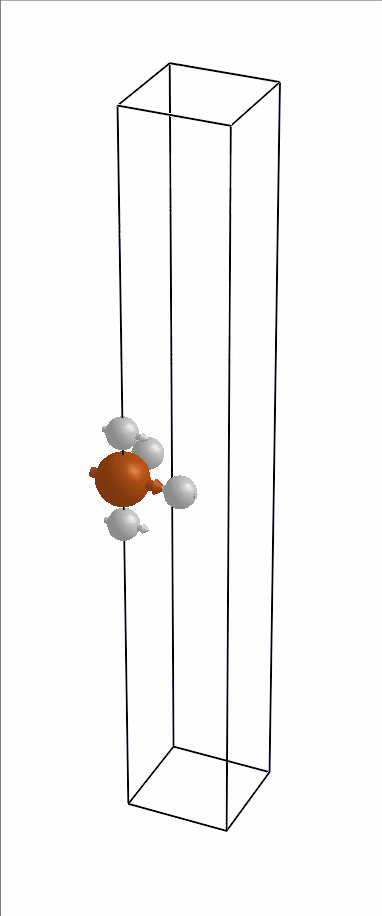

Supplement: Supplementary file 1 [file materials-17-02780-s001.zip › materials-3012524-supplementary/Supplementary Materials/Supplementary material S1 - Vibration modes of 2D TaF4/13.gif]

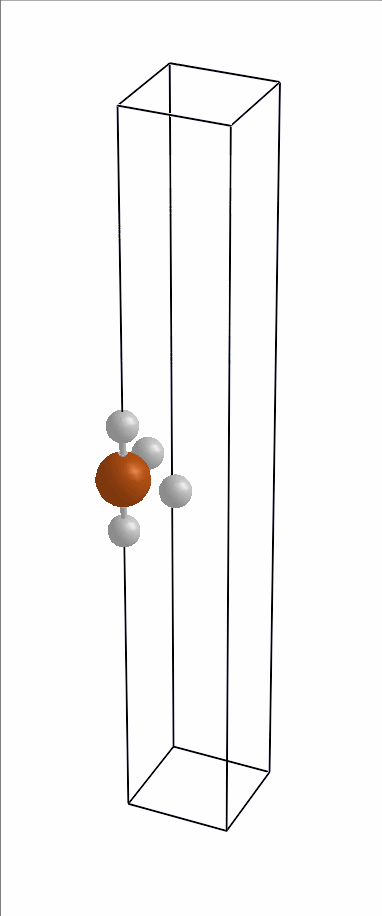

Supplement: Supplementary file 1 [file materials-17-02780-s001.zip › materials-3012524-supplementary/Supplementary Materials/Supplementary material S1 - Vibration modes of 2D TaF4/14.gif]

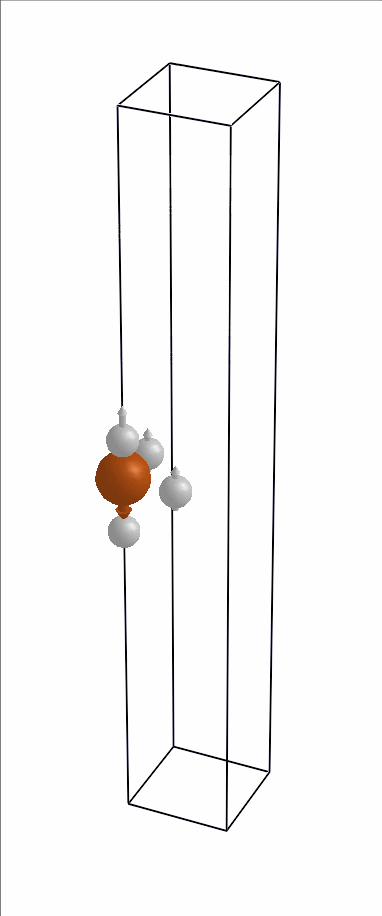

Supplement: Supplementary file 1 [file materials-17-02780-s001.zip › materials-3012524-supplementary/Supplementary Materials/Supplementary material S1 - Vibration modes of 2D TaF4/15.gif]
